# Functional Site Discovery From Incomplete Training Data: A Case Study With Nucleic Acid–Binding Proteins

**DOI:** 10.3389/fgene.2019.00729

**Published:** 2019-08-30

**Authors:** Wenchuan Wang, Robert Langlois, Marina Langlois, Georgi Z. Genchev, Xiaolei Wang, Hui Lu

**Affiliations:** ^1^SJTU-Yale Joint Center for Biostatistics and Data Science, Department of Bioinformatics and Biostatistics, College of Life Science and Biotechnology, Shanghai Jiao Tong University, Shanghai, Chinas; ^2^Department of Bioengineering and Department of Computer Science, University of Illinois at Chicago, Chicago, IL, United States; ^3^Bulgarian Institute for Genomics and Precision Medicine, Sofia, Bulgaria; ^4^Institute of Science and Technology for Brain-Inspired Intelligence, Fudan University, Shanghai, China; ^5^Center for Biomedical Informatics, Shanghai Children’s Hospital, Shanghai, China

**Keywords:** machine learning, protein sequence and structural analysis, multiple-instance learning, decision trees, semi supervised learning, protein function annotation, DNA binding proteins, RNA binding proteins

## Abstract

Function annotation efforts provide a foundation to our understanding of cellular processes and the functioning of the living cell. This motivates high-throughput computational methods to characterize new protein members of a particular function. Research work has focused on discriminative machine-learning methods, which promise to make efficient, *de novo* predictions of protein function. Furthermore, available function annotation exists predominantly for individual proteins rather than residues of which only a subset is necessary for the conveyance of a particular function. This limits discriminative approaches to predicting functions for which there is sufficient residue-level annotation, e.g., identification of DNA-binding proteins or where an excellent global representation can be divined. Complete understanding of the various functions of proteins requires discovery and functional annotation at the residue level. Herein, we cast this problem into the setting of multiple-instance learning, which only requires knowledge of the protein’s function yet identifies functionally relevant residues and need not rely on homology. We developed a new multiple-instance leaning algorithm derived from AdaBoost and benchmarked this algorithm against two well-studied protein function prediction tasks: annotating proteins that bind DNA and RNA. This algorithm outperforms certain previous approaches in annotating protein function while identifying functionally relevant residues involved in binding both DNA and RNA, and on one protein-DNA benchmark, it achieves near perfect classification.

## Introduction

Computational tools have become indispensable in guiding, analyzing, and simulating the mechanistic details underlying experimental studies. Recent innovations in high-throughput experiments for function discovery have provided sufficient data to model and understand the characteristics that govern specific function using machine-learning methods. Such methods have been used to address biological problems ranging from microarray analysis and its application in diagnosis, therapy decisions, and clinical testing (Juneau et al., 2014; Peterson et al., 2015; Shen et al., 2018); inter-disease relationships and similarities (Carson et al., 2017; Qin and Lu, 2018) image-based diagnostics (Mehta et al., 2017); predicting protein structural characteristics (Langlois and Lu, 2010a; Abbass and Nebel, 2015; Andreeva, 2016; Kashani-Amin et al., 2018) or clinically relevant discovery enabled by next-generation sequencing data of genomes and transcriptomes of diseased and normal cells (Gunaratne et al., 2012; Hayes and Kim, 2015; Gu et al., 2017; Liu et al., 2017; Gong et al., 2018; Liu et al., 2018).

High-throughput sequence and structural genomics projects have continued to outpace corresponding functional discovery projects producing a deluge of protein data, with only a fraction having some functional annotation. This annotation typically provides an indication of the general function but rarely, and when available—less reliably—provides mechanistic detail for a particular function. Systems biology research has focused on analyzing and predicting known interactions between proteins whereas pharmaceutical research requires greater knowledge in the mechanistic details of molecular function. Both efforts would benefit from machine-learning methods that can accurately classify protein function using the limited amount of training data available.

There are two approaches to the classification problem motivated by different statistical views: generative and discriminative learning. On one hand, the generative approach attempts to solve a more general problem i.e., modeling [*p*(*x,y*)] providing greater flexibility at the cost of computational complexity. In order to design an efficient generative algorithm, strong assumptions must be made; e.g., in sequence alignment, one makes the assumption that sequence similarity equals function similarity. On the other hand, discriminative classifiers attempt to find a direct mapping between the class label (*y*) and the input vectors (*x*). Since this approach solves the specific problem at hand, rather than a more general problem, discriminative approaches should be preferred to generative ones (Libbrecht and Noble, 2015). However, the fact remains that generative, sequence alignment techniques remain predominant in the face of recently developed discriminative approaches. So, why have these discriminative techniques not been more successful? The fundamental problem seems to be that research has focused on a single type of discriminate method, classification, which requires labeled training examples. Since protein function annotation data is limited, only a few functional groups such as nucleic acid–binding proteins provide sufficient labeled training data.

A number of discriminative techniques have been developed to deal with incomplete knowledge of the training data such as: semi-supervised learning (Chapelle et al., 2010), active learning (Reker and Schneider, 2015), positive and unlabeled learning (Bhardwaj et al., 2010), and multiple-instance learning (MIL) (Carbonneau et al., 2018). While the first three approaches have demonstrated that unlabeled training data can be used to improve learning, the last approach leverages additional information, i.e., labeled groupings of unlabeled data. In MIL, examples (also referred to as instances) are organized into groups called bags. The class label is associated with the bag rather than the instance; the bag is labeled positive if at least one instance in the bag is labeled positive; otherwise, the bag is labeled negative. Consider the functional site discovery problem: functional data usually pertains to the protein rather than to specific functional sites. Hence, in the MIL formulation, the protein is a labeled bag and the residues (or motifs or pockets) are the instances belonging to that protein/bag.

MIL was originally developed for handwritten digit recognition by Keeler et al. (1990) and was later popularized by Dietterich et al. (1997) to predict drug activity. It has subsequently been applied to a number of problem domains including context-based image retrieval (Maron and Lozano-Perez, 1998; Andrews et al., 2003a), protein super-family annotation (TrX proteins) (Scott et al.), and text categorization (Ray and Craven, 2005). A number of algorithms have been developed to solve MIL including convolutional neural networks (Keeler et al., 1990), axis parallel (Dietterich et al., 1997), support-vector machines (Doran and Ray, 2014), diverse density (Maron and Lozano-Perez, 1998), and standard binary classifiers (Ray and Craven, 2005).

MIL algorithm–based approaches have recently found increased use in the diagnosis of cancer (Li et al., 2015; Mercan et al., 2018; Yousefi et al., 2018), application in neurology for classification of brain abnormalities (Tong et al., 2014), and the prediction of phenotype from metagenomics data (Rahman et al., 2017) to name a few. Recent work has utilized MIL-based methods to predict major histocompatibility complex class II (MHC-II)–binding peptides (Xu et al., 2014) and transcription factor-DNA interaction (Gao et al., 2015; Gao and Ruan, 2017).

The boosting framework has also been conscripted to solve MIL problems. These approaches fall into two groups: modify the weak learner or modify the boosting cost function. That is, Auer and Ortner (2004) took the first approach by boosting a weak MIL-algorithm based on hyper-balls. Other algorithms have been developed using the second approach. For example, Andrews and Hofmann (2003b) used disjunctive logic programming (Lee and Grossmann, 2000) to create a boosting algorithm that achieves a large margin for at least one instance in each bag. Likewise, other groups (Xu and Frank, 2004; Viola et al., 2006) have used a derivation of the AnyBoost framework (Mason et al., 1999) to design an MIL cost function, which can be solved by numerical optimization.

Our work herein formulates the function prediction problem in the setting of MIL. In our approach, the function of a protein is identified through the discovery of key residue microenvironments that strongly signal the existence of a particular functional site. This method requires only two sets of example sequences or structures: one that has the function of interest and another that does not. We do not require knowledge of the functional sites yet this method automatically discovers such sites in order to predict the function of the protein. In the formulation of this approach, we predict function rather than superfamily assignment of a protein; moreover, we represent the protein by each residue’s microenvironment rather than by pre-calculated conserved motifs.

To solve this problem, we developed a novel boosting algorithm (Langlois, 2008) derived from the AdaBoost framework (Schapire and Singer, 1999) that efficiently and accurately identifies residue microenvironments that correspond to functional sites. We then benchmark this approach on two protein function assignment problems: the identification of DNA- and RNA-binding proteins. These proteins play an essential role in nearly every cellular process. A number of experimental (Cajone et al., 1989; Freeman et al., 1995; Chou et al., 2003; Buck and Lieb, 2004; Nutiu et al., 2011; Gordan et al., 2013) and computational (Bhardwaj et al., 2005; Szilagyi and Skolnick, 2006; Bhardwaj and Lu, 2007; Langlois et al., 2007; Tjong and Zhou, 2007; Gao and Skolnick, 2009; Langlois and Lu, 2010b; Weirauch et al., 2013; Xu et al., 2015) approaches have been developed to identify these proteins and their functional sites. Since DNA- and RNA-binding proteins provide a substantial number of labeled examples, e.g., residues known to bind DNA or RNA, these problems have been studied extensively thus presenting an excellent proof of concept for our approach.

## Results

We demonstrate the ability of an MIL algorithm to accurately predict the function of a protein using its constituent residues with two benchmark nucleic-acid binding datasets: DNA- and RNA-binding proteins. The characteristics of each dataset are summarized in **Table 1**. Both datasets have been used in previous studies to identify residues that bind DNA (Szilagyi and Skolnick, 2006; Langlois et al., 2007) and RNA (Terribilini et al., 2006; Langlois et al., 2007; Kumar et al., 2008). During training, each residue in a DNA-binding protein is considered DNA-binding and in a non-DNA-binding protein non-binding during training and cross-validation. Nevertheless, these residue-level labels are used for later evaluation of the algorithm on the residue level.

**Table 1:** Tabulates the number of proteins in both the DNA and RNA datasets.

	Total	Protein Positive	Negative	Total	Residue Positive	Negative
**DNA**	310	60	250	109,826	2,505	107,321
**RNA**	304	80	224	91,538	3,235	88,303

### Protein Function Annotation

We compare two learning algorithms to solve the MIL problem: AdaBoost and AdaBoost.C2MIL on decision trees. The first algorithm, AdaBoost on decision trees is a classification algorithm, which views MIL as a classification problem with positive class noise (Blum, 1998). While other classifiers have been extensively tested on MIL problems (Ray and Craven, 2005), AdaBoost on decision trees has not; this is due to its past poor performance on problems with mislabeled data (Schapire, 1999). The second algorithm AdaBoost.C2MIL is a modification of the original AdaBoost algorithm we developed specifically to handle MIL, which gives special treatment to instances (residues) in a positive bag (DNA-binding protein).

**Table 2** summarizes the performance of each algorithm in terms of area under the receiver operating characteristic (ROC) curve on the protein-level (first column), residue-level over the entire dataset (second column), and over just the DNA-binding proteins (third column). The protein-level results demonstrate the effectiveness of the proposed C2MIL variant over the standard AdaBoost algorithm where C2MIL outperforms AdaBoost by 5% on the DNA-binding task and by 6% on the RNA-binding task. The residue-level performance over the entire dataset is worse in both cases. However, this is due to the inclusion of residues from non-binding proteins, which skew the results. When considering the more pertinent case of only nucleic acid–binding proteins, the C2MIL algorithm outperforms AdaBoost in both cases: by almost 9% for the DNA-binding task and 3% for the RNA-binding task.

**Table 2:** Performance of algorithms in the multiple-instance learning (MIL) function prediction task—area under the receiver operating characteristic (ROC) curve.

		Protein	Residue (All)	Residue (-NA)
**DNA binding**	AdaBoost	90.3	84.4	63.2
	AdaBoost.C2MIL	95.8	82.7	72.1
**RNA binding**	AdaBoost	84.1	79.4	65.6
	AdaBoost.C2MIL	90.2	74.5	68.7

The performance over the DNA-binding set on the protein-level exceeds several previously published works. First, the performance of the C2MIL algorithm achieves 95.8% area under the ROC whereas the best previous result was 93% (Szilagyi and Skolnick, 2006) and 91.0% (Langlois and Lu, 2010b). At 85.0% specificity, C2MIL achieves 94.4% sensitivity compared to 89.0% (Szilagyi and Skolnick, 2006). At 95.0% specificity, Stawiski et al. (Stawiski et al., 2003) achieved 81.0% sensitivity while C2MIL 86.1% sensitivity. Finally, at 98% specificity, Langlois and Lu (Langlois and Lu, 2010b) achieved 48.1% sensitivity and C2MIL 70.8% sensitivity. Overall, C2MIL shows marked improvement in accurately predicting whether a protein binds DNA.

### Functional Site Prediction

Since no residue-level labels were given during training, i.e., the algorithm does not know which residues bind DNA or RNA, the performance of C2MIL is significantly less than the current best: 72% (**Table 1**) *versus* 83% (Langlois et al., 2007) in terms of area under the ROC. At the same time, the performance over the full dataset (both DNA-binding and non-binding proteins) is significantly better than over just the DNA-binding proteins: 82.7% area under the ROC (**Table 1**). This seems to indicate that non-binding residue environments or substructures on non-NA-binding proteins are easier to predict than corresponding ones on NA-binding proteins.

**Figure 1:**
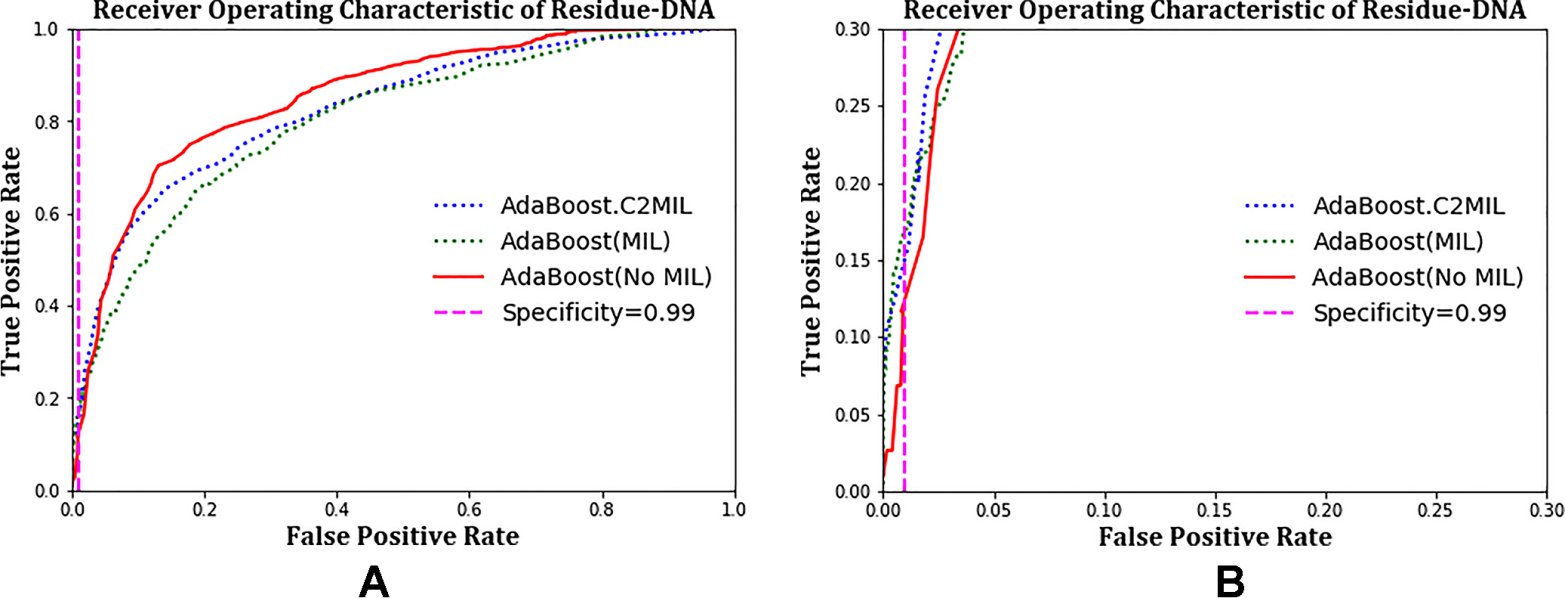
Comparison of learning tasks and algorithms on the residue-level over the entire dataset using a receiver operating characteristic curve: **(A)** entire curve and **(B)** zoomed on the 99% specificity.

**Figure 2:**
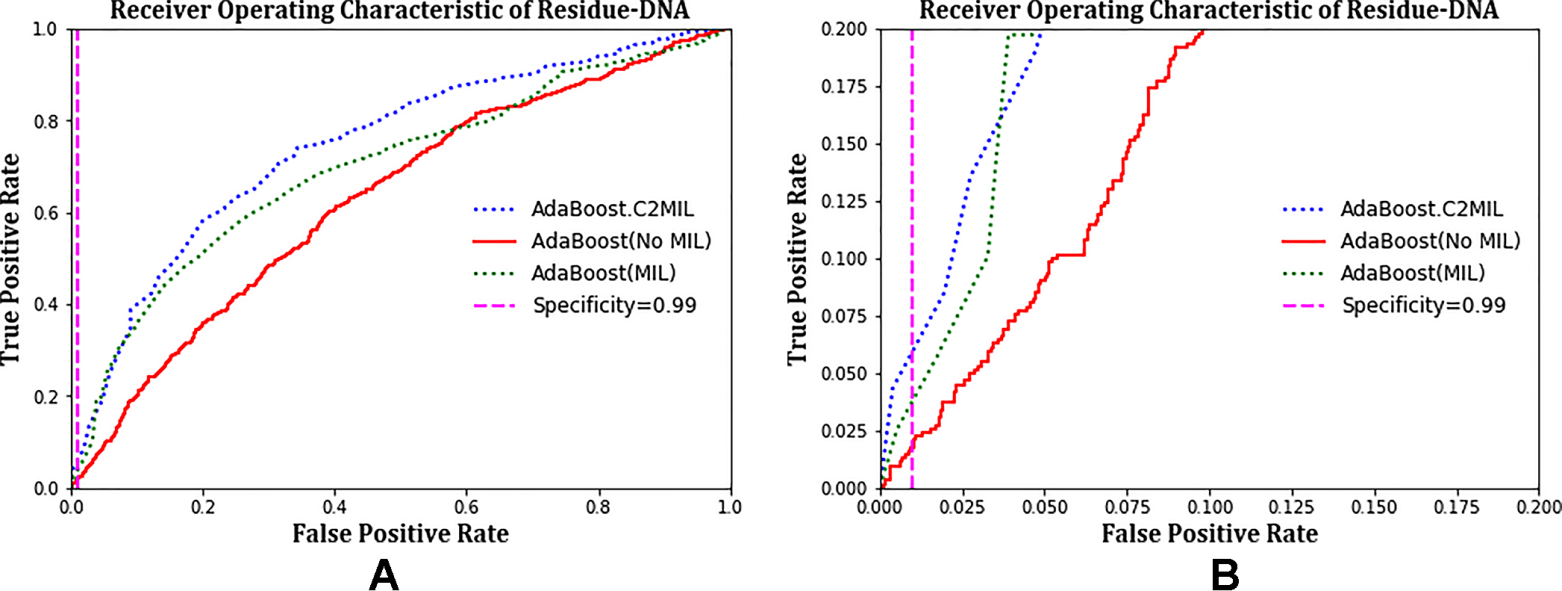
Comparison of learning tasks and algorithms on the residue-level over only DNA-binding proteins using a receiver operating characteristic curve: **(A)** entire curve and **(B)** zoomed on the 99% specificity.

The ROC plots in **Figure 1** and **Figure 2** compare the performance of C2MIL with the standard AdaBoost algorithm over the DNA-binding dataset. In **Figure 1A**, both algorithms cross several times with no clear winner. However, at low false-positive rates (**Figure 1B**), C2MIL dominates the standard AdaBoost providing an explanation for C2MIL’s better performance on the protein level. Since only a single residue predicted positive means the entire bag is positive, this is the important region on the residue-level ROC curve.

The ROC plots in **Figure 2** compare the performance of C2MIL with the standard AdaBoost algorithm over the residues from only DNA-binding proteins. This evaluation follows that of other DNA-binding papers (Langlois et al., 2007). On this task, C2MIL dominates the standard AdaBoost algorithm over the entire range of the ROC plot. As the protein-level results indicate, C2MIL finds at least one residue microenvironment that strongly indicates a given protein is DNA binding. Moreover, these instance-level results demonstrate that not many residues fit the bill given the rather low sensitivity at low false-positive rates.

### Trends in Residue-Level Prediction

**Figure 3:**
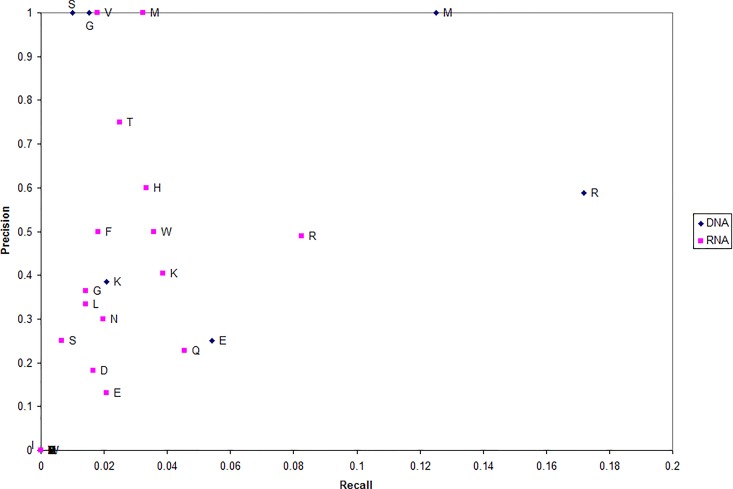
Precision and recall for each nucleic acid (NA)-binding protein residue type.

To better understand the residue microenvironments that characterize NA-binding proteins, we plot each type of residue which has been correctly predicted DNA binding in terms of recall and precision (**Figure 3**). Precision measures the fraction of residues predicted NA binding that are actually DNA binding (in blue) and RNA binding (in red). Recall measures the fraction of NA-binding residues correctly predicted NA binding.

The first trend evident in **Figure 3** is that far more residues can be used to predict a protein RNA binding (red) as opposed to DNA binding (blue). This suggests that more residues are involved in protein-RNA interactions than protein-DNA. Second, arginine is unsurprising the dominant residue predicted for both NA-binding proteins.

Third, DNA-binding proteins can unexpectedly be well characterized by microenvironments centered on either serine (S) or glycine (G) with a precision of 1.0; e.g., every serine predicted as DNA binding actually was DNA binding. While previous works have suggested glycine (specifically its content) as more correlated with the non-binding set (Bhardwaj et al., 2005; Szilagyi and Skolnick, 2006; Langlois et al., 2007), it has been observed that glycine can make non-specific interactions with DNA (Luscombe and Thornton, 2002) and that glycine-rich linkers are critical to regulatory protein function (Singh et al., 2014).

Fourth, a set of RNA-binding proteins can be accurately characterized by microenvironment centered on either valine (V) or methionine (M) with a precision of 1.0. These residues as well as histidine and threonine have been found important experimentally. Threonine has been shown to make specific interactions with both splice sites (Colwill et al., 1996; Zhang and Fuller, 2003) and rRNA (Clemens et al., 1993). Likewise, histidine has been found important for specificity (Hake et al., 1998) and valine makes unique interactions with viral RNA (Pinck et al., 1970).

Note that, in proteins predicted DNA/RNA binding, these four residues (V, M, S, and G) provide a rough location the NA-binding site each protein. This demonstrates that the MIL-algorithm identifies DNA-/RNA-binding proteins based on residue important to their function.

## Discussion

Conventional approaches that apply machine learning to function prediction have relied on a global representation of the sequence or structure, or a local representation of a residue’s environment on a target protein. In the first case, only examples of known proteins with a particular function are required whereas the second case requires knowing the location of the active sites. Our proposed approach is similar to sequence alignment techniques in that we require only knowing the function of a particular protein and not the functional residues. Moreover, similar to sequence analysis techniques, it identifies a subset of probable functional residues. Nevertheless, our proposed algorithm does not require sequence similarity or homology to be effective (unlike sequence analysis techniques).

In this work, we demonstrate the ability of our MIL algorithm–based approach to identify potential binding sites and, through the presence of such a site, the function of the protein. This is done without knowledge of the binding sites during the training process. Essentially, one can both identify the function of and locate a binding site on a test protein without knowing, during the training process, the location of such sites. One can view MIL over structure-based features as sub-structure analysis where were consider a sliding window along the amino acid chain throughout the structure. Thus, a user only requires knowledge of the protein function, not the particular site, yet the resulting learning algorithm can predict both.

The proposed approach also has several advantages over traditional homology-based methods:

- Does not rely on finding a similar structure/sequence
- Discovers functional sites with little prior knowledge

Our method does not require homologous sequences or structures; instead, it relies on physio-chemical characteristics in combination with (when available) structural features. It can also be applied to problems where knowledge of the functional site is limited. We also provide an analysis of MIL algorithms on the instance level. In some previously published MIL works, the authors evaluate their algorithms on the bag-level since instance-level labels are either unavailable or unreasonably expensive to obtain.

This works establishes the ability of our MIL algorithm–based method to outperform classification in discriminating RNA- or DNA-binding proteins from non-binding proteins. The success of this approach relies on the better representation of function permitted by the MIL problem formulation. Instead of representing the protein sequence or structure by some global representation, the MIL approach allows the entire protein to be decomposed into potential functional units and discovers which unit actually performs the function. Developing a feature encoding for a single functional unit is far easier than for the entire protein sequence or structure.

While multiple-instance (MI) learning has several advantages over classification, it remains a harder learning problem in that the learning algorithm does not have access to instance-level labels. Nevertheless, the experiments clearly show that the proposed MI learner does not perform substantially worse when identifying residues that bind DNA or RNA. Indeed, these results compare favorably with the current state-of-the-art in residue classification.

There are several limitations to the present work. First, we do not limit the algorithm to only sequence information; yet, this will provide the primary source of data for this application. Second, this work does not consider open conformations, e.g., proteins not in complex with DNA. Since the current set of features does not require the exact residue orientation, this may not be a significant limitation. Third, it does not incorporate known binding residues; such residues can provide more information regarding these residues. This problem can be remedied through the application of active MIL (Zhang et al., 2008). Fourth, this algorithm would utilize and would benefit from far larger datasets such as sequences in the UniProt (Leinonen et al., 2004) database. Finally, the analysis of the important residues was just a first-order approximation to the potential wealth of information this technique can glean from both sequence and structural data.

## Materials and Methods

### Dataset

There are two stringent benchmark datasets used for DNA- and RNA-binding protein prediction tasks. The first set is 60 DNA-binding proteins and 250 non-DNA-binding proteins derived by Liu et al. (2014) and later used by Shen et al. (2017) and Wei et al. (2017) (Supplementary Table 1). The second set is 80 RNA-binding proteins and 224 non-RNA-binding proteins used by Miao and Westhof (Miao and Westhof, 2015) and Paz et al. (2016)(Supplementary Table 2). The two datasets are both acquired from the Protein Data Bank, and short sequences (less than 50 amino acids) and sequences containing the consecutive character “X” have been removed. To eliminate the redundancy and homology bias that likely leads to overestimated performance, it removes sequences with ≥25% pairwise sequence identity to any other sequences in the dataset using the program CD-HIT.

**Figure 4:**
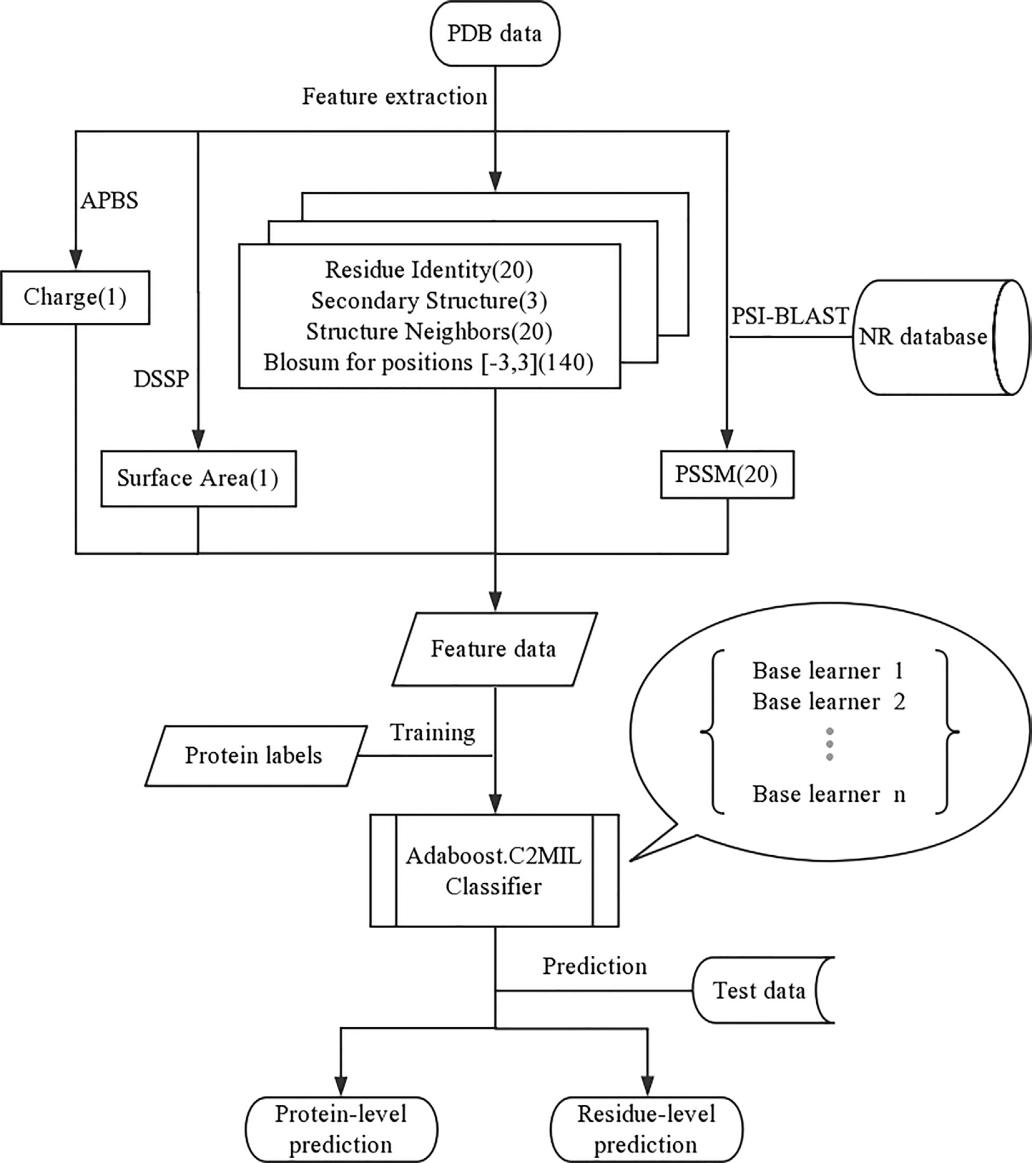
Overall framework the proposed AdaBoost.C2MIL method and feature extraction.

Each residue in the protein is represented using the following features (feature count within parenthesis) (**Figure 4**):

- Residue identity (Bhardwaj et al., 2010)
- Secondary structure (Shen et al., 2018)
- Structure neighbors (Bhardwaj et al., 2010)
- PSSM for residue at that position (Bhardwaj et al., 2010)
- BLOSUM for positions $-3…3$ (140)
- Properties: Charge, Surface Area (Juneau et al., 2014)

The residue identifier is a 20-dimensional vector where the residue type is indicated by a non-zero value in the corresponding column. Likewise, there is a corresponding secondary structure identifier feature vector. The structure neighbors count the frequency of each residue type within 3 Å (measured heavy atom to heavy atom). The PSSM feature scores the conservation of this residue position. The BLOSUM window also estimates the residue conservation within a window around the specific residue. Finally, the properties of charge and surface area are estimated for each residue. For more details concerning the feature representation, see Langlois et al. (2007).

### Algorithm

The Adaptive Boosting (AdaBoost) algorithm transforms a weak classifier *L*(·) into a strong ensemble classifier *H*(·)(44). AdaBoost proves most effective with decision trees as the weak classifier (often referred to as “the best off-the-shelf classifier”) and has one tunable parameter: the number of boosting iterations (*T*). Rather than the general boosting framework as in prior work (Mason et al., 1999), we propose to modify the AdaBoost algorithm itself to reduce MIL to importance-weighted classification.

**Equation 1:** Proposed AdaBoost.C2MIL Algorithm

**Given**: {(*X*_1_,y_1_) …(*X* *_n_* *,y* *_n_*)} where : ${\text{X}}_{i}=\left\{{\vec{x}}_{1}\ldots {\vec{x}}_{n_{i}}\right\}\text{ and }y_{i}\in \{-1,1\}\text{ and }{\vec{x}}_{j}\in X$ Reorganize dataset such each negative bag contains one instance **Initialize**: $w_{t=1,i}=\frac{1}{n},i=1\;\ldots \;n$ **For** *t* = 1 …*T* 1. Map dataset to instance level: $\hat{\text{D}}=\left({\vec{x}}_{i,j},y_{i},\frac{w_{i}}{n_{i}}\right)$ 2. Train weak classifier on instance-level dataset $L(\hat{D})$ 3. Get confidence-rated instance-level hypothesis ${\hat{h}}_{t}:X\to ℜ$ 4. $\hat{p}=\frac{1}{1+\exp(-{\hat{h}}_{t})}$ 5. Get weak bag-level hypothesis: $h_{t}(X_{i})=\frac{\Sigma_{j}{\hat{p}}_{t}({\vec{x}}_{i,j})\mathrm{sgn}\left[{\hat{h}}_{t}\left({\vec{x}}_{i,j}\right)\right]}{\Sigma_{j}{\hat{p}}_{t}({\vec{x}}_{i,j})}$ 6. $\varepsilon_{t}=\sum_{\mathrm{sgn}[h_{t}(X_{i})]\neq y_{i}}w_{t,i}$ 7. $\alpha_{t}=\frac{1}{2}\ln\left(\frac{1-\varepsilon_{t}}{\varepsilon_{t}}\right)$ 8. $w_{t+1,i}=w_{t,i}\cdot \exp(-\alpha_{t}\mathrm{sgn}[h_{t}(X_{i})]\mathrm{sgn}[y_{i}])/Z_{t},i=1\;\ldots n$ **Output:** $H({\vec{x}}_{i,j})=\sum_{1}^{T}\alpha_{t}{\hat{h}}_{t}({\vec{x}}_{i,j})\text{ Instance-level prediction}$ $H(X_{i})=\sum_{1}^{T}\alpha_{t}h_{t}(X_{i})\text{ Bag-level prediction}$

The proposed algorithm, AdaBoost.C2MIL, is outlined in Equation 1. The first step in the algorithm is to set up the dataset. It starts by reorganizing the dataset such that each negative instance becomes its own bag while the positive instances remain grouped in their original bags. Note that, since we know each instance in a negative bag must be negative, this step does not disregard useful information. It then sets up a uniform weighted distribution on the bag level. Since each negative instance is a bag, it has its own weight whereas instances in a positive bag share a single weight.

The second step, within the for loop, starts by mapping the MIL dataset to a classification dataset where every instance in a positive bag is labeled positive, and the weight is split uniformly among the instances. Next, the algorithm trains a weak classifier (*L*) over the current distribution of the dataset, which gives confidence-rated hypothesis ${\hat{h}}_{t}$. The confidence-rated prediction follows (Schapire and Singer, 1999) and can be converted to a probability using the sigmoid function. Finally, for positive bags (and negative bags during evaluation), the bag-level prediction is a summation of the instance-level predictions (step 5).

The rest of the algorithm follows AdaBoost on the bag level. First, the algorithm estimates the bag-level error and then calculates the step size α. This step size is then used to increase the weight on incorrectly predicted bags and decrease on correctly predicted.

The output of the ensemble multiple-instance learner acts on both the bag and instance level. Each classifier contributes to the prediction of an instance whereas the bag-level prediction is made by the equation in step 5.

### Experiments

The overall framework of our experiment is represented in **Figure 4**. The AdaBoost algorithm requires a weak learner and, as a weak learner, the decision tree works well across the board; we use a custom implementation with a top-down (Kearns and Mansour, 1996) impurity function for confidence-rated boosting. The algorithms, metrics, and graphs used in this work were generated using python. The performance is measured using 5-fold stratified cross-validation. And code is available at https://github.com/WintrumWang/AdaBoost.C2MIL.

## Author Contributions

RL and HL designed the project, WW and RL performed the project, ML and GG helped in method development and manuscript writing, XW participated in the computation. All authors approved the writing.

## Funding

This work is partially supported by National Key R&D Program of China 2018YFC0910500, the Neil Shen’s SJTU Medical Research Fund, SJTU-Yale Collaborative Research Seed Fund; NSFC 31728012, Science and Technology Commission of Shanghai Municipality (STCSM) grant 17DZ 22512000, Shanghai Municipal Science and Technology Major Project (No. 2018SHZDZX01), LCNBI and ZJLab. RL acknowledges the support from NIH training grant T32 HL 07692.

## Conflict of Interest Statement

The authors declare that the research was conducted in the absence of any commercial or financial relationships that could be construed as a potential conflict of interest.

## Supplementary Material

Supplementary Table 1 The DNA-binding protein benchmark dataset.

Supplementary Table 2 The RNA-binding protein benchmark dataset.
